# Clinical Holistic Medicine: Psychodynamic Short-Time Therapy Complemented with Bodywork. A Clinical Follow-Up Study of 109 Patients

**DOI:** 10.1100/tsw.2006.352

**Published:** 2006-10-30

**Authors:** Søren Ventegodt, Suzette Thegler, Tove Andreasen, Flemming Struve, Lars Enevoldsen, Laila Bassaine, Margrethe Torp, Joav Merrick

**Affiliations:** ^1^ Quality of Life Research Center, Teglgårdstræde 4-8, DK-1452 Copenhagen K, Denmark; ^2^ Research Clinic for Holistic Medicine, Copenhagen, Denmark; ^3^ Nordic School of Holistic Medicine, Copenhagen, Denmark; ^4^ Scandinavian Foundation for Holistic Medicine, Sandvika, Norway; ^5^ Interuniversity College, Graz, Austria; ^6^ Zusman Child Development Center Soroka University Medical Center Ben Gurion University of the Negev, Beer-Sheva, Israel; ^7^ National Institute of Child Health and Human Development, Jerusalem, Israel; ^8^ Office of the Medical Director Division for Mental Retardation Ministry of Social Affairs, Jerusalem, Israel

**Keywords:** holistic medicine and health, complementary and alternative medicine, clinic effect

## Abstract

This is a study of 109 patients who attended the Research Clinic for Holistic Medicine in Copenhagen during the 2004–2006 period, grouped according to the symptoms they presented with. Every new patient was asked to answer a 10-question composite questionnaire containing QOL1, QOL5, and four questions on ability to function socially, ability to function sexually, ability to love, and ability to work, rated on a 5-point Likert scale, on initial contact and after 1–3 months, when the patient had received about five treatments, the patient was asked to complete the questionnaire again, and finally again after 1 year. All had been to their general practitioner first with their problems and 30% had been in psychological/psychiatric treatment before. The patients were treated with short-time psychodynamic therapy (less than 40 sessions) including bodywork when necessary. More than half the patients had a bad or very bad self-assessed mental health before treatment, but after treatment only 15% reported a bad or very bad mental health (p < 0.001). Most had a complex of mental, somatic, existential, and sexual problems. Of the patients, 69.72% did the retest after treatment. We conclude that clinical holistic medicine was able to help the majority of these patients, even when patients had not been sufficiently helped by drugs, psychiatry, or psychology before. We found that outcome of therapy was not connected with severity of initial condition, but probably with the former experience of treatment. If psychiatric or psychological treatment had already failed, the patients were more difficult to help. The Square Curve Paradigm was used to document a large, immediate and lasting effect of the therapy.

### INTRODUCTION

A number of studies have documented the efficiency of psychodynamic short-time therapy[1,2,3], but the problem has been that the immediate clinical efficiency has been modest compared to the effect of drugs, especially with severe mental problems and diseases. The effect of psychotherapy on somatic diseases has also been disputed[4], which has led to a number of new intervention strategies collected under the umbrella of “holistic medicine”. Holistic medicine is a popular and blossoming field, and a search on www.pubmed.gov shows almost 5,000 scientific papers on holistic medicine with hundreds of new papers added yearly.

It is important to notice that 400,000 Danes used alternative/holistic medicine in 1990, but in 2000 it had increased to 800,000[5] and is expected to be 1,600,000 in 2010. In 2020, it is estimated that alternative/holistic medicine utilization will further increase, as it has done in the U.S. already, where it is generally believed that there are now more CAM consultations than biomedical consultations. In spite of all this activity, the effect of holistic medicine in general is still not clear. This is primarily because the term now refers to hundreds of treatment systems focused on “the whole patient” and not primarily on the symptoms or the disease as it is in normal practice in today’s mainstream biomedicine, pharmacological medicine. It seems that what works in holistic medicine is basically that the physician or therapist induces healing on an existential level, called salutogenesis by Aaron Antonovsky (1923–1994)[6,7], by creating a deep shift in the consciousness of the patient towards the more positive and constructive.

The reason for the medical efficiency of such a shift towards positive attitudes and behaviors is that consciousness seems to be the primary determinant of global quality of life (QOL), health, and ability[8]. Because of the appreciation in the causal power of consciousness, many physicians and therapists are now focusing on this important shift in the patient’s consciousness as their primary goal in treatment (to improve QOL, health, and ability of the patient). This focus has caused the emerging field of “clinical holistic medicine”.

The present study aims to explore the effectiveness of *clinical holistic medicine* on a variety of patients that have not been sufficiently helped by their biomedically (pharmacologically) oriented general practitioner, or felt insufficiently helped by their psychiatrist and/or other health professionals in the biomedically oriented public health system of Denmark.

#### The Basis for Their Treatment and Evaluation

The treatment is psychodynamic regressive therapy based on Sigmund Freud[9] and Carl Gustav Jung[10,11] (and to a smaller extent Wilhelm Reich[12], Alexander Lowen[13], Frits Perls[14], Stanislav Grof[15,16], Aaron Antonovsky[6,7], Marion Rosen[17]), allowing the patients to integrate old emotionally painful material (traumas or gestalts) primarily from early childhood. A number of the patients regressed spontaneously all the way “back into their mothers womb”, when the advanced holistic medical tools were taken into use[18].

About 18 months ago, the staff at the Research Clinic for Holistic Medicine in Copenhagen decided to use the square curve paradigm[19] as the primary tool for research on effects of treatment and quality control in the clinic. The procedure was quite simple, as every new patient was asked to answer a 10-question composite questionnaire containing QOL1[20], QOL5[20], and four questions on ability to function socially, ability to function sexually, ability to love, and ability to work; rated on a 5-point Likert scale (see below).

After 1–3 months[19], when the patient had received about five treatments, the patient was asked to complete the questionnaire again to see if the treatment actually helped the patient. This procedure was performed again after 1 year in order to investigate if the therapeutic gain was permanent[19].

Most of the patients that came to the clinic had already been to their general physician and many of them had also seen specialists, psychiatrists, psychologists, or others in order to get help. They came to our clinic because they were not satisfied with the results of their earlier treatment(s). They received an initial conversation about the study they were entering and about what results could be expected in their individual case without any promise of improvement on their health, since QOL improvement was the direct goal. Subsequent reading of relevant patient information, books, and scientific papers allowed them to decide which treatment to choose and finally, after closing a therapeutic contract, they entered therapy. This model was constructed on our present scientific knowledge of integrative medicine[21,22,23, 24,25,26,27] and clinical holistic experience[28,29,30,31,32,33,34,35,36,37,38,39,40,41,42,43,44,45, 46,47,48,49,50,51,52,53,54,55,56,57,58,59,60,61,62,63,64,65,66,67,68,69,70,71,72,73,74].

### Principles of Treatment

There were four core principles for the treatment:

1. Induce healing of the whole existence of the patient and not only his/her body or mind[75,76,77,78,79,80,81,82,83]. The healing often included goals like recovering purpose and meaning of life by improving existential coherence[6,7] and ability to love, understand, and function sexually[6,7,10,11,12,13,21,22,23,24,25,26,27].
2. Adding as many resources to the patient as possible[18,21,22,23,24,25,26,27], as the primary reason for originally repressing the emotionally charged material was lack of resources — love, understanding, empathy, respect, care, acceptance, and acknowledgment — to mention a few of the many needs of the little child. The principle was also to use the minimal intervention necessary by first using conversational therapy, then additional philosophical exercises if needed, then adding bodywork or, if needed, adding role play, group therapy, and finally when necessary in a few cases, referring to a psychiatrist for psychopharmacologic intervention[18]. If the patient was in somatic or psychiatric treatment already at the beginning of the therapy, this treatment was continued with support from the holistic therapist.
3. Using the similarity principle that seems to be a fundamental principle for all holistic healing[21,22,23,24,25,26,27]. The similarity principle is based on the belief that what made the person sick originally will make the patient well again, when given in the right, therapeutic dose. This principle often leads to dramatic events in the therapy and to efficient and fast healing, but seems to send the patient into a number of crises that must be handled professionally[72]. The scientific background for a radical and fast healing using the similarity principle is analyzed[21,22,23,24,25,26,27].
4. Using Hering's Law of Cure[21,22,23,24,25,26,27] to support the patient in going once again through all the disturbances and diseases, in reverse order, that brought the patient to where he or she is now. Other important axioms of Hering's Law of Cure is that the disease goes from more- to less-important organs, goes from the inside out, and goes from upside down. The scientific rationale for the last three axioms are less clear than for the first: The patient must go back through his/her timeline in order to integrate all the states and experiences he/she has met on his/her way to disease. Going back in time is normally done though spontaneous regression in holistic existential therapy.

## METHODS

The protocol for the research project "Quality of Life and Causes of Disease I and II" was originally accepted by the Danish Scientifically Ethical Committee. It had two major steps: first (1990–1997) asking 11,000 Danes over 3,000 questionnaires on quality of life, health, and hundreds of other life factors to understand how to intervene and help patients improve their quality of life and health. After completing this analysis, the next level (1997–2010) integrated this new knowledge into the established traditional knowledge on psychodynamic therapy. The purpose of the project was to see if treating the patient with this expanded system would be efficient compared to traditional psychoanalysis and other psychodynamic therapy known only to help a limited number of patients with a limited number of diseases. This is a slow process and intervention can span over several years and hundreds of treatments. The data collected pointed to the importance of including philosophy of life- and bodywork in the treatment, which was done experimentally in 1997 with fair results in pilot studies, justifying a more extensive study starting in 2000 with now more than 500 patients included. An important part of the study was the establishment of a training facility for physicians, nurses, occupational therapists, physiotherapists, and other health professionals in order to provide the training necessary to deliver the treatment according to a specific standard. This resulted in the creation of the Nordic School of Holistic Medicine in 2002. All therapists participating in the present study have been trained at this facility.

The second step included the development of general strategies for helping patients with the most common diseases, so all therapists at the clinic could treat all patients as uniformly as possible. The therapists were instructed and trained to "diagnose" the patient, not using traditional somatic or psychiatric diagnoses, but rather more holistic categories that seemed relevant to general practice with holistic medicine. The therapist was supervised while treating the patients. This project initiated in 1990 at the University Hospital of Copenhagen (Rigshospitalet) (1990–1994 at that medical center and later moved to another location) was made possible through substantial funding of more than four million EURO in total from two large not-for-profit foundations and contributions from more than 300 private companies and private donors.

A total of 109 patients were included in this part of the study running for the 2004–2006 period, and they were grouped according to the symptoms they presented with and some patients being in more than one group. The patients received the QOL10 questionnaire containing the validated QOL1 and QOL5 questionnaires, and their answers were discussed with a physician to ensure that the patients knew how to fill it in correctly and secure the validity and quality of the research data. One important problem in this phase was that many patients did not completely realize their sad condition before they had had some hours of therapy. Because of that fact, the second QOL measure (T2 after 1–3 months) was often lower than the initial QOL measure at the beginning of the therapy (T1). This is still an important measurement because we need to balance the effect of patients feeling bad and rating low at the beginning of the therapy, since presumably they would rate themselves a little better also without therapy. Another conservative feature in the square curve paradigm is that the patient is to confront old emotional pains in holistic existential therapy and, therefore, T2 is often low.

As the square curve paradigm is designed to run without a control group as it detects immediate and permanent improvements in the patient's state of being, quality of life and health status, an appreciation of the conservative nature of this paradigm is important for acknowledging its validity. Interestingly, the square curve paradigm runs nicely with 10–20 patients[19] and, in these situations, the measurement will be statistically significant only when the improvement of quality of life, health, and/or ability is large enough to have clinical significance also.

Criteria of inclusion were that the patient had entered the program after we started measuring with QOL5 in 2004 and they had to have been to at least three sessions.

### RESULTS

#### Clinical Holistic Medical Treatment in General

A total of 109 patients entered the study and 76 (69.72%) did the retest after the treatment. The patients that dropped out of therapy are all included in the study.

- 65.1% of the patients had somatic problems; these patients received an average 12.5 sessions at a price of EURO 998.40.
- 59.6% of the patients had sexual problems; these patients received an average 14.8 sessions at a price of EURO 1,188.00.

- 71.6% of the patients had existential problems; these patients received an average 14.5 sessions at a price of EURO 1,160.00.
- 51.4% of the patients had mental problems; these patients received an average 11.7 sessions at a price of EURO 938.40.

Most patients, thus, had a complex disease pattern with many mental, somatic, existential, and sexual problems, most often over 10 problems on their self-reported problem list, when therapy started. There is an element of judgment in categorizing the patients according to the four categories above. If a patient has a stomach problem, this could be seen as a physical problem (pain), a mental problem (somatized anxiety), a sexual problem (sexual blockages), or an existential problem (i.e., as a blockage of the hara-center/2$^{nd}$ chakra). The treating physician has made an "objective" judgment of how to categorize the patient.

Of the 109 patients entering the study, 28 (25.7%) terminated the treatment before the therapist found that the treatment was completed. We speculate that patients that are not benefiting from the therapy are more motivated to drop out. Further research is needed to illuminate this important question.

We sent the patient a questionnaire by mail and called them to interview them by the telephone if they did not reply. The response rate of the retest was 69.72%, which we find acceptable. Nonresponders might be more negative towards the treatment than responders, but late responders did not answer much differently from early responders, indicating that this problem was not likely to be very important.

Two patients out of the 500 we have treated in the last 5 years have complained that they temporarily felt worse after the end of the holistic treatment than before they started. Both had disrupted the treatment themselves just before the time when the therapist believed they would have had their breakthrough in the treatment.

No permanent side effects were found from the treatment on any of the 500 patients that until now have received the "new formula" of holistic existential therapy, but about 20 patients had temporary crises during the treatment. The crises were analyzed to be of three types and relate to each other as described in a paper[72]. When we analyzed and learned about such "spiritual" crisis, we were able to predict them and use them in our care for the patients and hereby in practice, eliminating this problem.

The QOL1[20] measures self-assessed quality of life, and this might be the single-most important measure to use in holistic medicine, as this measure indicates the total state of your being. Happily, we have found that most patients responded positively to holistic treatment by this measure (see Table 1). The QOL5[20] measure goes into much more detail with the patient's subjective feeling of well-being and health, both mental and physical, and in this measure, most of the patient groups were also improving through the treatment (see Table 1). QOL10 included four measures of ability to function focusing on social ability, ability to love, sexual ability, and working ability. QOL 10 was constructed the same way as QOL1 and QOL5, but has not yet been scientifically validated. We also found that most patients improved in this measure. Submeasures of the analyses are listed below:

1. Self-assessed physical health on a 5-point Likert scale
2. Self-assessed mental health on a 5-point Likert scale
3. Health in general; average of self-assessed physical health and self-assessed mental health
4. Self-assessed relationship with self on a 5-point Likert scale
5. Self-assessed relationship with friends on a 5-point Likert scale
6. Self-assessed relationship with partner on a 5-point Likert scale
7. Quality of life (QOL5)[20]
8. Quality of relationships (average of three above)
9. Self-assessed ability to love on a 5-point Likert scale
10. Self-assessed sexual ability on a 5-point Likert scale
11. Self-assessed social ability on a 5-point Likert scale

**TABLE 1**
**Effect of Holistic Medical Treatment of 109 Patients at the Research Clinic for Holistic Medicine 2004–2006**

**Paired Samples**

| | | Paired Differences | | | | | t | df | Sig. (2-tailed) |
|---|---|---|---|---|---|---|---|---|---|
| | | | | | 95% Confidence Interval of the Difference | | | | |
| | | Mean | Std. Deviation | Std. Error Mean | Lower | Upper | | | |
| Pair 1 | Physical health before-after | ,3421 | 1,00070 | ,11479 | ,1134 | ,5708 | 2,980 | 75 | ,004 |
| Pair 2 | Mental health before-after | ,9737 | 1,16589 | ,13374 | ,7073 | 1,2401 | 7,281 | 75 | ,001 |
| Pair 3 | Total health before-after | ,6467 | ,88826 | ,10257 | ,4423 | ,8510 | 6,305 | 74 | ,001 |
| Pair 4 | Relations before-after | ,6930 | ,98397 | ,11287 | ,4681 | ,9178 | 6,140 | 75 | ,001 |
| Pair 5 | Ability before-after | ,6467 | ,92186 | ,10645 | ,4346 | ,8588 | 6,075 | 74 | ,001 |
| Pair 6 | Quality of life before-after | ,6847 | ,82233 | ,09559 | ,4942 | ,8752 | 7,162 | 73 | ,001 |
| Pair 7 | Health-QOL-Ability bef-after | ,6840 | ,76904 | ,09063 | ,5033 | ,8647 | 7,547 | 71 | ,001 |

12. Self-assessed working ability on a 5-point Likert scale
13. Average ability (mean of four above)
14. Self-assessed quality of life on a 5-point Likert scale (QOL1)[20]
15. Average of health, quality of life, and ability (QOL10)

Table 1 shows an overview of the results from the survey.

### Results from This Study

Table 1 gives the major findings of the study. Figs. 1 and 2 show the general self-assessed health (average of somatic and mental self-assessed health measured on two 5-point Likert scales from “very good” to “very bad”[20]), before and after the treatment. Before the treatment, about 50% of the patients felt ill (below 3 on the scale) and about 25% experienced a compromised health (3 on the scale: neither good nor bad). More patients than that had either a bad self-assessed physical or mental health, but here we are looking on the average of both, the health in general. After the treatment, only 20% felt ill (below 3 on the scale) and 15 % had a compromised health (3 on the scale). The transformation of about 40% of the patients from ill to healthy (from below 3 to above 3 on the scale) corresponds to a statistical finding that the group as a whole has gone from a mean of 3.12 to a mean of 2.51 (see Figs. 1 and 2).

When we look at self-reported experience about the therapy, we found that 68% of the patients felt they have been helped (t3, n = 57).

The same positive developments were seen in the measure quality of life as measured with the validated questionnaire QOL5[20]. Here the patients also jumped from below 3 (a mean of 3.2) to above 3 (a mean of 2.53) on the scale (see Figs. 3 and 4).

We found the same positive development in the patients’ general ability to function (see Figs. 5 and 6).

A standard deviation in all three measures of 0.7–0.8 tells us that most patients actually benefited from the treatment in all three dimensions. This is the proof of salutogenesis according to the theory[6,7]. The improvement of about one step in the three scales corresponds to a very large clinical effect on the patients.

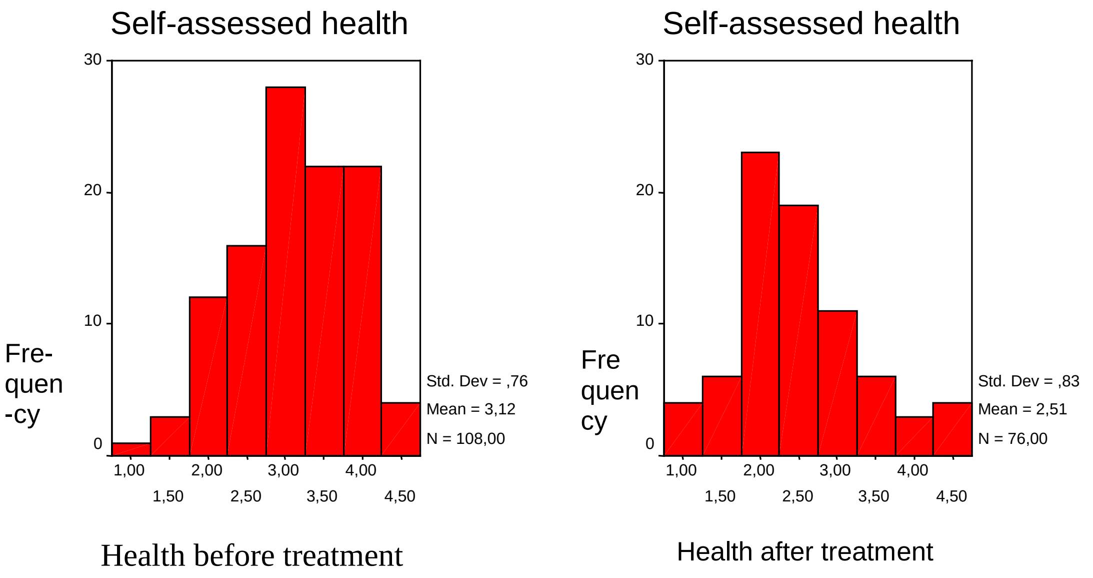

**FIGURES 1 and 2.** Healing of the patient's life (salutogenesis) documented through the improvement of self-assessed physical and mental health, before and after the treatment with clinical holistic medicine.

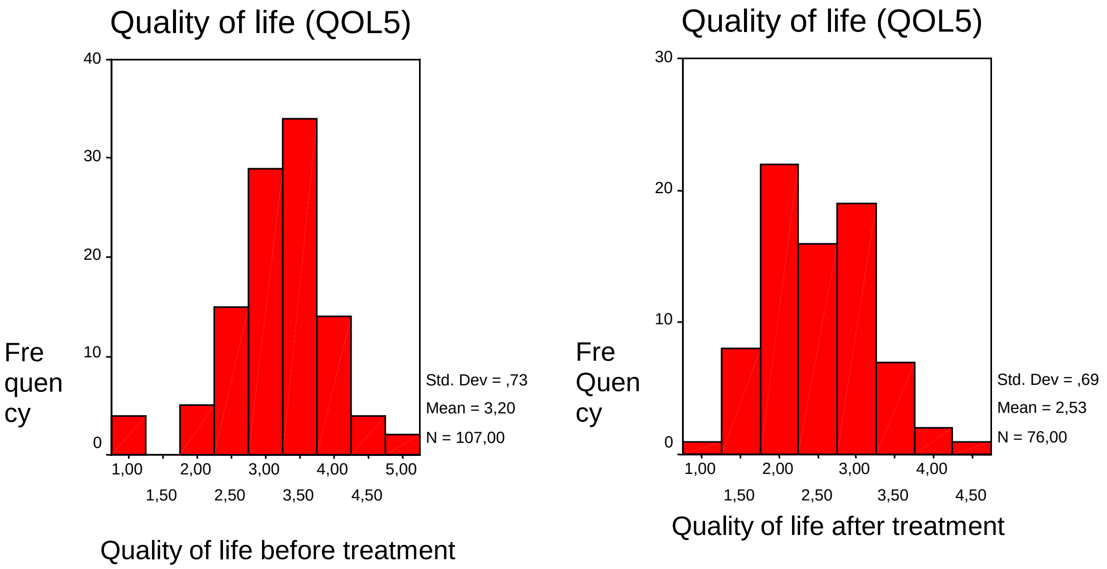

**FIGURES 3 and 4.** Healing of the patient's life (salutogenesis) documented through the improvement of the patient's self-assessed quality of life as measured with QOL5[20], before and after the treatment with clinical holistic medicine.

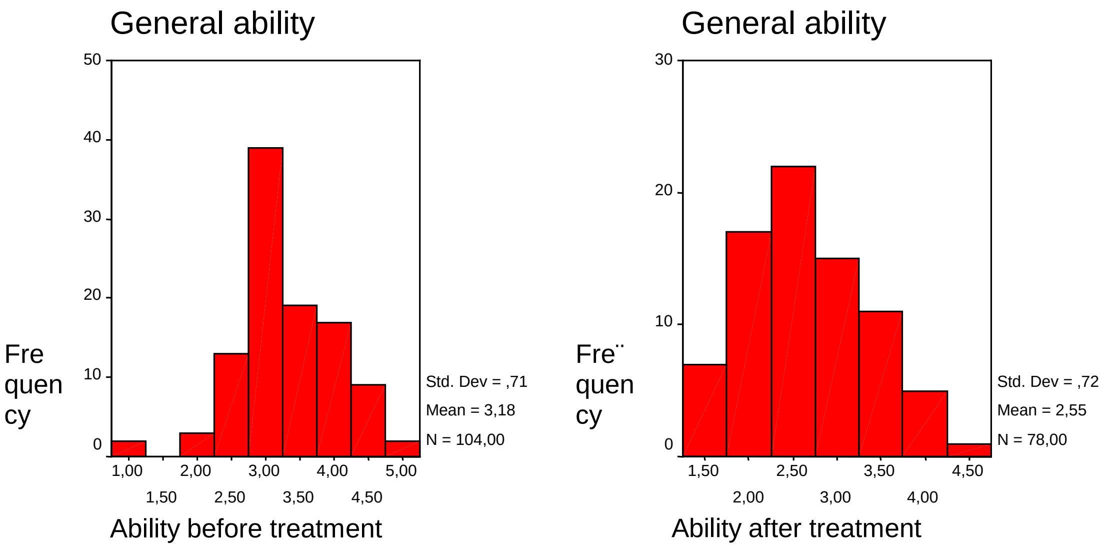

**FIGURES 5 and 6.** Healing of the patient's life (salutogenesis) documented through the improvement of the patient's self-assessed ability to work, love, function socially and sexually, before and after the treatment with clinical holistic medicine.

### The Square Curve Paradigm Test for Lasting Results

In existential healing, the results must be lasting. To test this, we conducted a prospective study in accordance with the "square curve paradigm"[19]. We asked the patients before the treatment (T1), immediately after starting (1–3 months) (T2), and then again about 1 year after completing the treatment (12 months after T2, the second measuring) (T3). From this time series (see Table 2 and Figs. 7, 8, and 9), which included more than 50% of the patients, it can be concluded that the effect of the treatment on the patient's health is actually lasting. As demanded by the square curve paradigm, the effect must not be lost during time; we found a statistically significant improvement from T1 to T2, but no significant drop from T2 to T3 (see Table 3 and Fig. 10, which illustrated the development of the physical health with the whole group of patients). We can conclude that the healing seemed to be large and permanent, and included all major areas of life.

#### TABLE 2
### Subjective Health Significantly Improved with Clinical Holistic Medicine

**Paired Samples**

| | | Paired | | | | | t | df | Sig. (2- |
|---|---|---|---|---|---|---|---|---|---|
| | | Mea | Std. | Std. Mea | 95% Interval of Differenc Lowe | Uppe | | | |
| Pair | Health T1 – Health T2 | ,554 | ,7857 | ,1059 | ,342 | ,767 | 5,23 | 54 | ,01 |
| Pair | Health T1 – Health T3 | ,705 | ,9084 | ,1213 | ,462 | ,948 | 5,81 | 55 | ,01 |
| Pair | Health T2 – Health T3 | ,166 | ,6210 | ,1035 | - | ,376 | 1,61 | 35 | ,11 |

Health = (Q1 + Q2)/2; T1, T2, and T3 are the times of measuring.

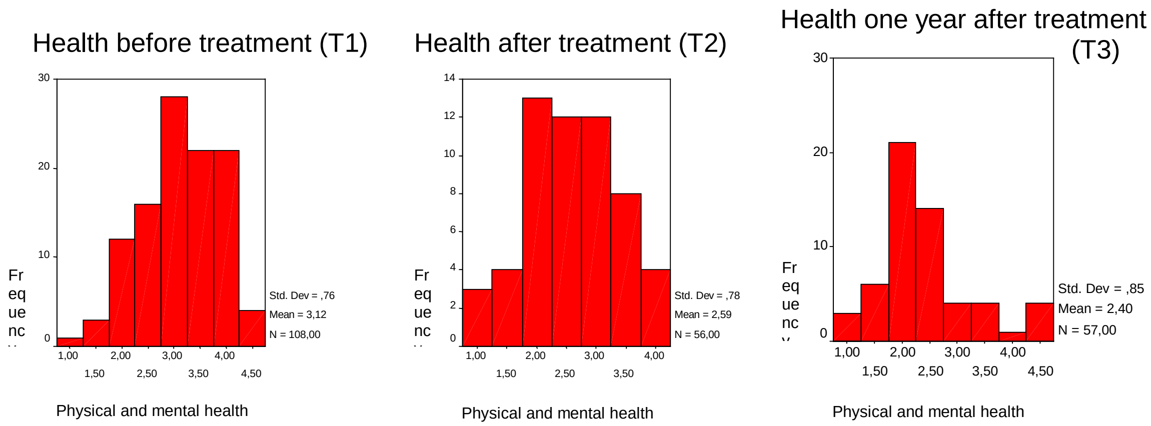

**FIGURES 7, 8, and 9.** Permanent healing of the patient's health (holistic/existential healing) documented through the three measures of the square curve paradigm: before treatment (T1), immediately after (T2), and 1 year after (T3). It can be seen that the improvement seems to continue and, when completed, we expect the patient's state of being to be fine and stable.

### TABLE 3
## Self-Assessed Physical Health Significantly Improved with Clinical Holistic Medicine

**Paired Samples Test**

| | | Paired Differences | | | | | | | |
|---|---|---|---|---|---|---|---|---|---|
| | | | | | 95% Confidence Interval of the Difference | | | | |
| | | Mean | Std. Deviation | Std. Error Mean | Lower | Upper | t | df | Sig. (2-tailed) |
| Pair 1 | T1Q1 - T2Q1 | ,3214 | ,87609 | ,11707 | ,0868 | ,5560 | 2,746 | 55 | ,008 |

**Paired Samples Test**

| | | Paired Differences | | | | | | | |
|---|---|---|---|---|---|---|---|---|---|
| | | | | | 95% Confidence Interval of the Difference | | | | |
| | | Mean | Std. Deviation | Std. Error Mean | Lower | Upper | t | df | Sig. (2-tailed) |
| Pair 1 | T1Q1 - T3Q1 | ,3214 | 1,09722 | ,14662 | ,0276 | ,6153 | 2,192 | 55 | ,033 |
| Pair 2 | T2Q1 - T3Q1 | ,1667 | ,77460 | ,12910 | -,0954 | ,4288 | 1,291 | 35 | ,205 |

QOL5, Q1 = self-assessed physical health; T1, T2, and T3 are the times of measuring.

The time series of quality of life (see Table 4) measured by QOL5 documents permanent improvement of quality of life (Figs. 11, 12, and 13).

We have one more measure of quality of life, which is self-assessed global quality of life (QOL1[20]) — "How would you rate your quality of life?" — rated on a 5-point Likert scale. We found the same tendency in the time series (Figs. 14, 15, and 16); the patients go from a mean of 3.4 to 2.4, and a standard deviation about one showing that most of the patients are improving.

The shift of a whole step in the Likert scale for the whole group of patients over only 1 year is quite remarkable. This is done with about 20 sessions of therapy. Most patients entered the clinic feeling really bad about their life, and after 1 year they feel fine. This is a very large clinical significance. Looking at the ability to function, we again found the same positive development (see Table 5 and Figs. 17, 18, and 19).

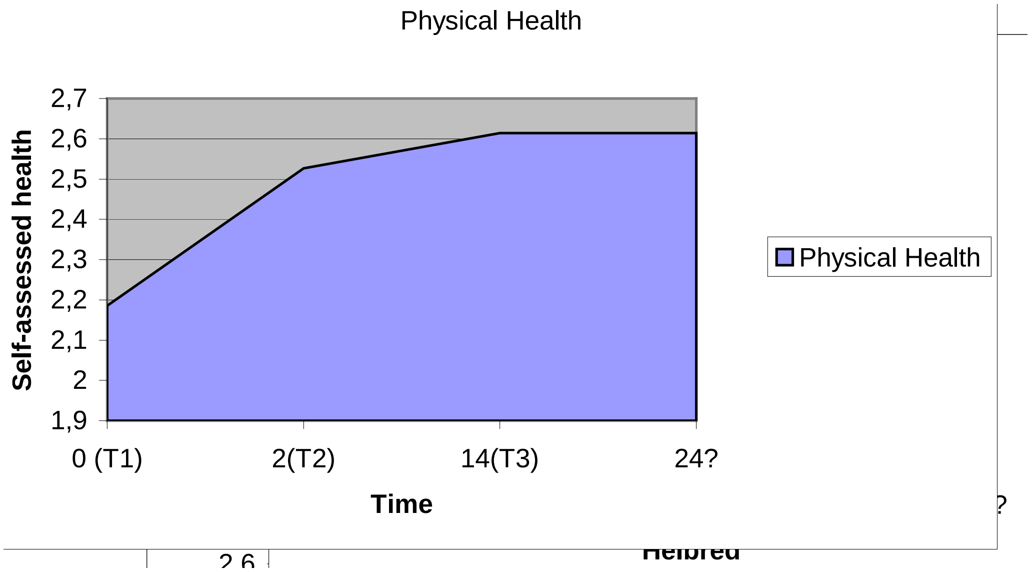

**FIGURE 10.** The almost perfect square curve[19] for physical health documenting self-assessed physical healing with clinical holistic medicine. The patients were measured with QOL 5[20] before treatment (T1), immediately after (T2), and 1 year after (T3). Time is in months. (Please notice the inverted scale here).

### TABLE 4
## Quality of Life as Measured with the Validated Questionnaire QOL5 Significantly Improved with Clinical Holistic Medicine

**Paired Samples Test**

| | | Paired | | | | | | | |
|---|---|---|---|---|---|---|---|---|---|
| | | | | | 95% Interval of Difference | | | | |
| | | Mean | Std. | Std. Mean | Lower | Upper | t | df | Sig. (2- |
| Pair 1 | QOL5_T1 - QOL5_T2 | ,4938 | ,73399 | ,09988 | ,2935 | ,6942 | 4,944 | 53 | ,001 |
| Pair 2 | QOL5_T1 - QOL5_T3 | ,7970 | ,81314 | ,10964 | ,5771 | 1,0168 | 7,269 | 54 | ,001 |
| Pair 3 | QOL5_T2 - QOL5_T3 | ,2963 | ,62502 | ,10417 | ,0848 | ,5078 | 2,844 | 35 | ,007 |

T1, T2, and T3 are the times of measuring.

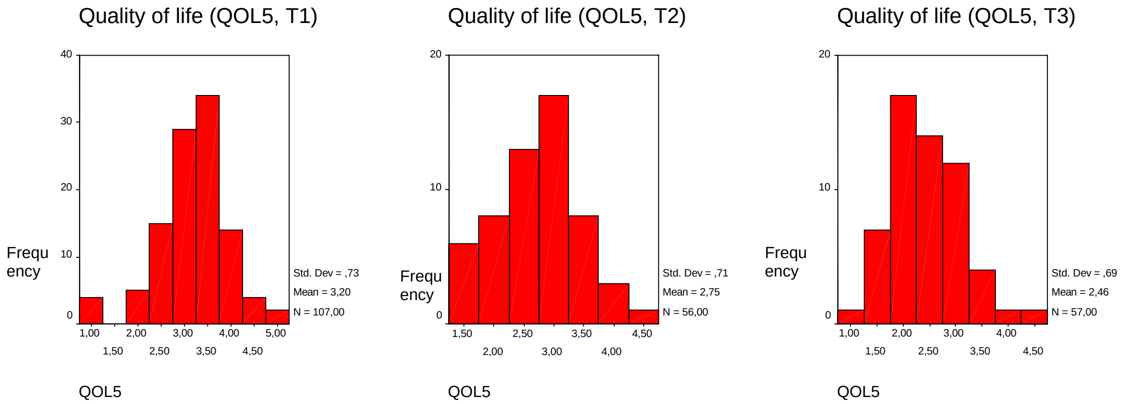

**FIGURES 11, 12, and 13.** Permanent healing of the patient's quality of life (QOL5) documented through the three measures of the square curve paradigm: before treatment (T1), immediately after (T2), and 1 year after (T3). It is seen that the improvement seems to continue and, when completed, we expect the patient's state of being to be fine and stable

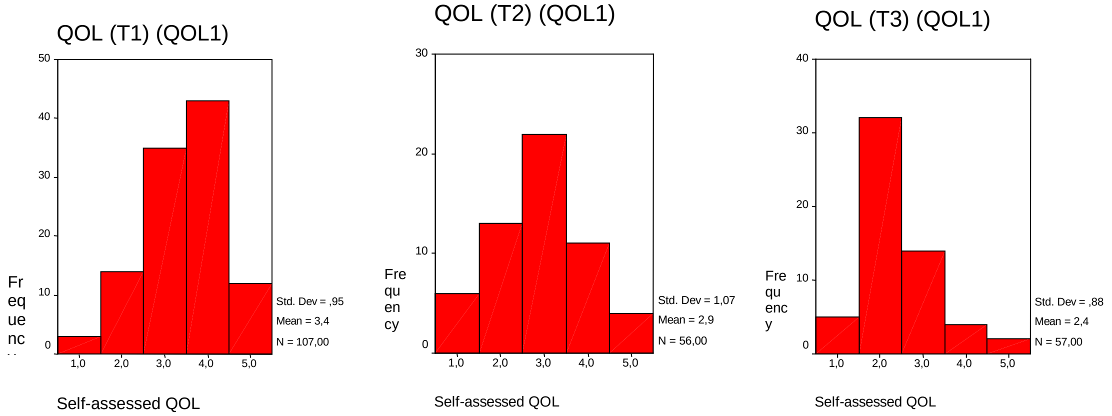

**FIGURES 14, 15, and 16.** Permanent healing of the patient's self-assessed, global quality of life (QOL1) documented through the three measures of the square curve paradigm: before treatment (T1), immediately after (T2), and 1 year after (T3). It is seen that the improvement seems to continue and, when completed, we expect the patient's state of being to be fine and stable.

### TABLE 5
## Ability to Function (Work, Social, Love, and Sex) as Measured with Four Questions on a 5-point Likert Scale Significantly Improved with Clinical Holistic Medicine

**Paired Samples**

| | | Paired | | | | | | | |
|---|---|---|---|---|---|---|---|---|---|
| | | | | | 95% Interval of Differenc | | | | |
| | | Mea | Std. | Std. Mea | Lower | Uppe | t | df | Sig. (2- |
| Pair | Ability T1-T2 | ,3818 | ,8591 | ,1158 | ,1496 | ,6141 | 3,296 | 54 | ,002 |
| Pair | Ability T1-T3 | ,7909 | ,8316 | ,1121 | ,5661 | 1,015 | 7,053 | 54 | ,001 |
| Pair | Ability T2 T3 | ,1875 | ,6718 | ,1119 | -,0398 | ,4148 | 1,675 | 35 | ,103 |

T1, T2, and T3 are the times of measuring.

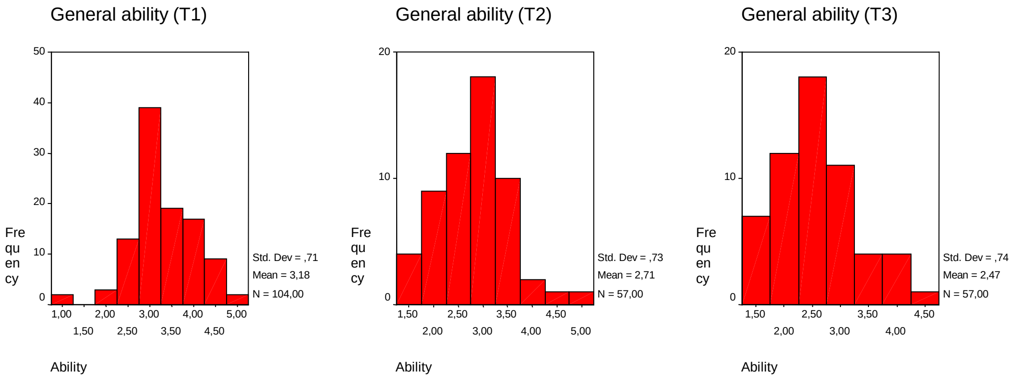

**FIGURES 17, 18, and 19.** Permanent healing of the patient's ability to function (sex, love, work, and social) documented through the three measures of the square curve paradigm: before treatment (T1), immediately after (T2), and 1 year after (T3). It is seen that the improvement seems to continue and, when completed, we expect the patient's state of being to be fine and stable.

If we take an integrative measure, QOL10, which is a mean of self-assessed physical and self-assessed mental health, self-assessed QOL, and self-assessed ability to function (sex, love, work, and social), we can more precisely see the effect of the holistic healing/salutogenesis (see Table 6 and Figs. 20, 21, and 22).

### TABLE 6
## Health, Quality of Life, and Ability All Combined in the Measure QOL10 Significantly Improved with Clinical Holistic Medicine

**Paired Samples**

| | Paired | | | | | t | df | Sig. (2- |
|---|---|---|---|---|---|---|---|---|
| | | | | 95% Interval of Differenc | | | | |
| | Mea | Std. | Std. Mea | Lower | Uppe | | | |
| Pair QOL10 T1 – T2 | ,464 | ,7266 | ,0998 | ,264 | ,665 | 4,65 | 52 | ,01 |
| Pair QOL10 T1 – T3 | ,792 | ,7038 | ,0957 | ,600 | ,984 | 8,27 | 53 | ,01 |
| Pair QOL10 T2 – T3 | ,241 | ,6099 | ,1016 | ,035 | ,447 | 2,37 | 35 | ,02 |

T1, T2, and T3 are the times of measuring.

The difference from a mean of 3.2 to 2.47 documents a completed existential healing with this group of patients. Many of the patients seemingly shifted from being unhappy, ill, and poorly functioning to being happy, healthy, and able. The effect is highly significant clinically. The effect has been reached with most of the patients having only 20 sessions of holistic, existential therapy. The relatively low degree of participation in the long series gives us some uncertainty of how the whole group of patients actually developed. The time series seem to document that half of the patients at least benefited and did so dramatically. To learn about the effect of the treatment, we need to go to the before-after test including 72.8% of the patients. This degree of participation seems to be fair and these results more reliable.

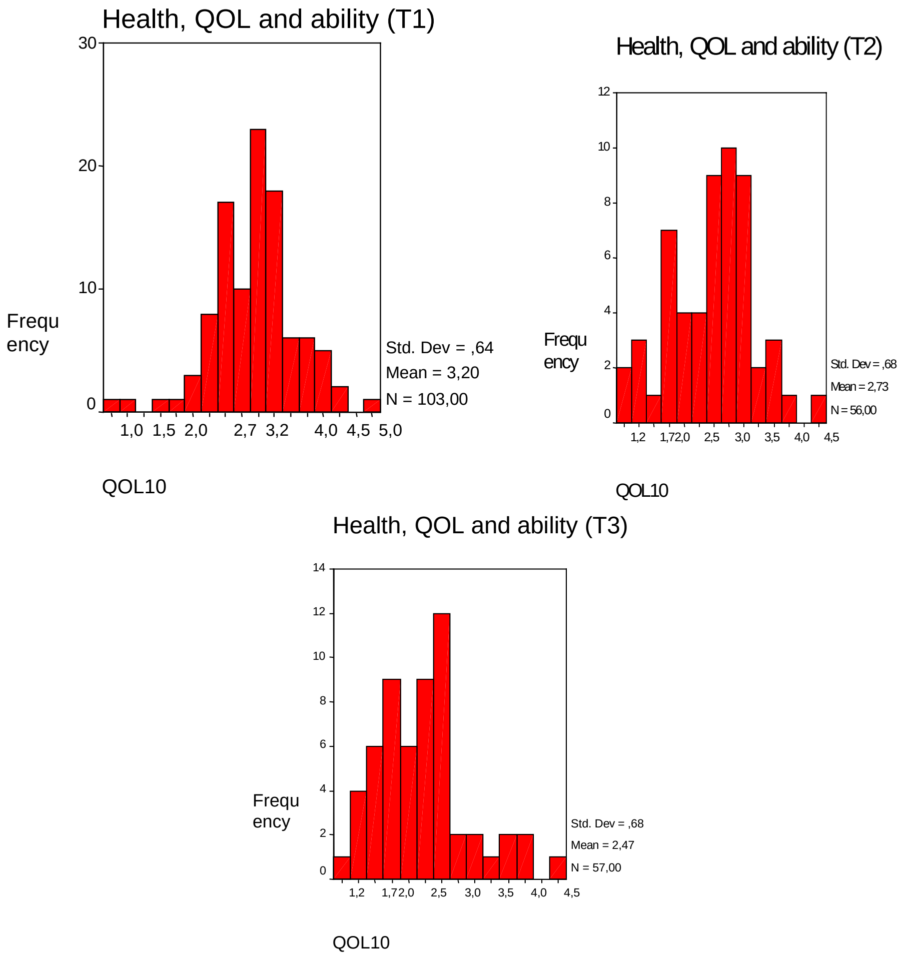

**FIGURES 20, 21, and 22.** Permanent healing of the patient's life (health, quality of life, and ability to function (sex, love, work, and social) documented through the three measures of the square curve paradigm: before treatment (T1), immediately after (T2), and 1 year after (T3). It is seen that the improvement seems to continue and, when completed, we expect the patient's state of being to be fine and stable. (Significance, T-test, paired samples: T1-T2: P< 0.01, T1-T3: P<0.01, and T2-T3: P=0.04).

### Mental Illness

Most patients came to our clinic with severe mental disturbances, evaluating their own mental health to be poor or very poor (4 or 5 on the 5-point Likert Scale of QOL5). Table 7 and Figs. 23, 24, and 25 show the The difference in mental health between T1 and T2 (before and after treatment) was not likely to be caused by anything else other than this treatment (cmp. square curve paradigm[19]). We believe that the patients were involving themselves in a lot of different events that also would help them to heal, because during the therapy, they learned the basic idea of holistic healing. Mental illness also has a periodical tendency (i.e., depression), so much of the improvement over a year might just be this kind of fluctuation. Still, every second patient who entered the clinic seemed to be mentally ill and after a year, only a fraction of these patients were still suffering from mental problems.

**TABLE 7**
**Self-Assessed Mental Health Significantly Improved with Clinical Holistic Medicine**

**Paired Samples**

| | | Paired | | | | | | | |
|---|---|---|---|---|---|---|---|---|---|
| | | | | | 95% Interval of Differenc | | | | |
| | | Mea | Std. | Std. Mea | Lower | Upper | t | df | Sig. (2- |
| Pair | Q2T1- Q2T2 | ,8000 | 1,1925 | ,16081 | ,4776 | 1,1224 | 4,975 | 54 | .001 |
| Pair | Q2T1- Q2T3 | 1,0893 | 1,0833 | ,14477 | ,7992 | 1,3794 | 7,524 | 55 | .001 |
| Pair | Q2T2- Q2T2 | ,1667 | ,91026 | ,15171 | -,1413 | ,4747 | 1,099 | 35 | .279 |

QOL5, Q2 = self-assessed mental health; T1, T2, and T3 are the times of measuring.

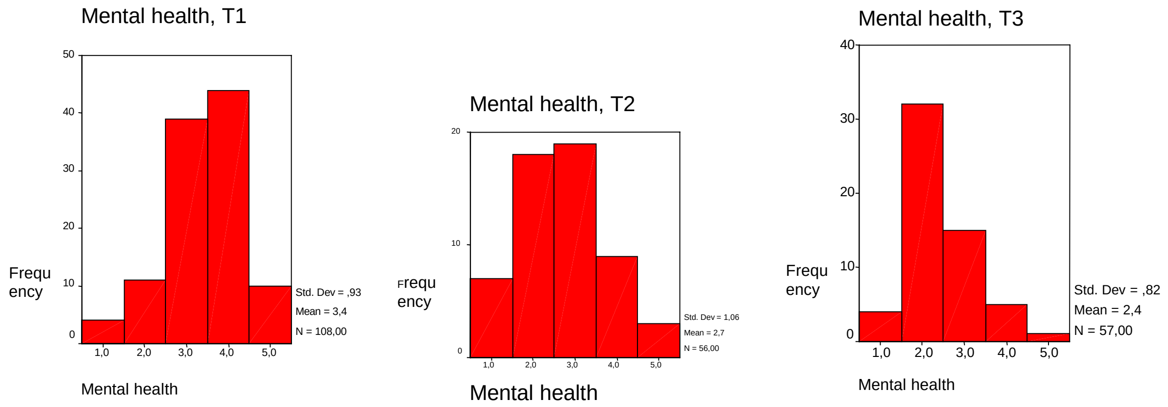

**FIGURES 23, 24, and 25.** Permanent healing (salutogenesis) of the mentally ill patient, documented through the three measures of the square curve paradigm: before treatment (T1), immediately after (T2), and 1 year after (T3). It is seen that the improvement seems to continue and, when completed, we expect the patient's state of being to be fine and stable.

### Patients Treated after Psychiatric, Psychological or Psychopharmacological Treatment Failed

Most patients entered our clinic after experiencing not being sufficiently helped by the regular Danish health system, which is free of charge for the patient (national health service). In our private clinic, they have to pay for the intensive short-time treatment, an average of about EURO 1,000 for 15 sessions. The patient with a long history of mental illness is difficult and, during our study period, we had 34 such patients. Results from their treatment are shown in Figs. 26 and 27.

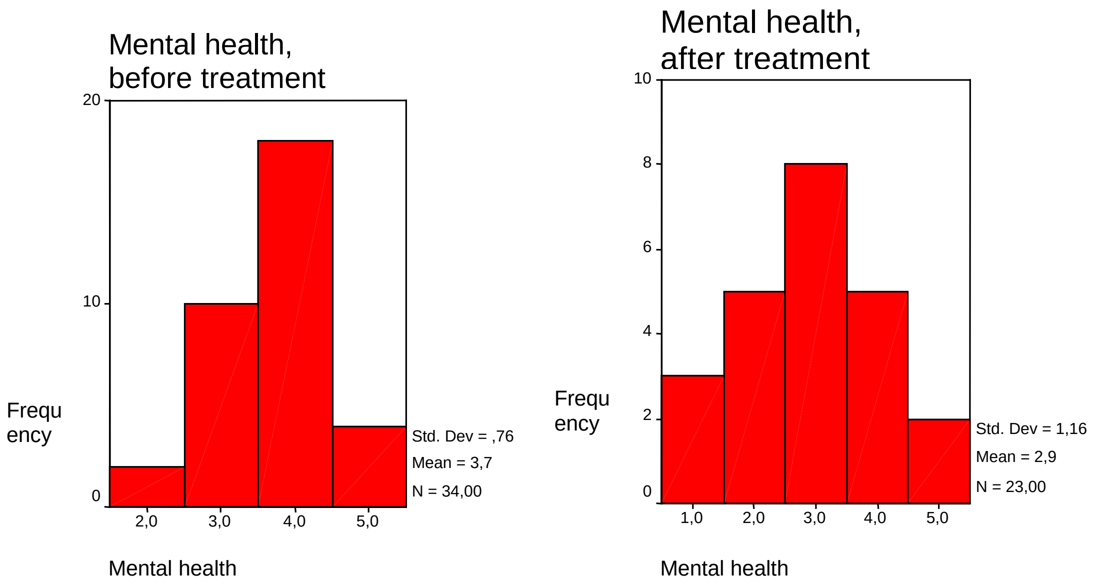

**FIGURES 26 and 27**. Holistic healing of 34 patients with a long history of mental problems. Most of the patients were rating their own mental health as "bad" before treatment; after treatment, most of the patients rated their mental health as "neither good nor bad". The improvement was from a mean of 3.7 to a mean of 2.9.

At the initial stage, about 60% of these patients rated their mental health as "bad" or "very bad", but after a little more than a year of treatment, 25% rated their mental health as bad or very bad. That the patients had been treated earlier with no satisfactory effect does not seem to be a hindrance for the holistic therapy to work. It is not that they start out much worse than the rest of the patients, but there seems to be a tendency that these patients, maybe because many of them already had a psychiatric diagnosis, believed less in holistic healing and more in the sad idea of being chronically mentally ill. If you do not believe that you can be healed, then it is easy to find yourself demotivated when it comes to struggling to get well again.

Table 8 documents the statistical significance of this result using paired t-test.

### DISCUSSION

It is important to stress that when clinical holistic medicine is focused on the effect of changing consciousness of the patient, which is also what placebo does, it has no meaning to test against placebo. To randomize a patient group and test against the patients that do not receive treatment will also affect the consciousness of both the winners and the losers of the randomization, so this cannot be done either. The only way to learn if an intervention on a patient's consciousness is successful seems to actually document that the treatment works; that is, if the patient is clinically significantly and permanently helped by the treatment. That is exactly what the square curve paradigm tests for.

The use of standard pharmacological methods in the area of complementary medicine is difficult due to the issue of placebo and the problem of placebo-controlling holistic medicine simply cannot be solved in our opinion[84], which in fact is not necessary either. Using the classical curve of healing known both by Hippocrates[75] and Hahneman[85], which we call the square curve created by sudden healing, we can test if a cure works. From our clinical experience and also from this study, we believe that holistic medicine most definitely does work, no matter if the patient has a somatic, mental, existential, or sexual problem.

**TABLE 8**
**Mental Health Significantly Improved with Clinical Holistic Medicine, Even When Former Psychiatric/Psychological Treatment Failed**

**Paired Samples**

| | | Paired | | | | | | | |
|---|---|---|---|---|---|---|---|---|---|
| | | | | | 95% Interval of Differen | | | | |
| Pair 1 | Mental health before - after | Mea | Std. | Std. Mea | Lowe | Uppe | t | df | Sig. (2- |
| | | ,818 | 1,1396 | ,2429 | ,312 | 1,323 | 3,36 | 21 | ,01 |

Paired t-test before intervention, contra after intervention.

The most important problem of this kind of intervention on the patient's consciousness is that we could fear that it is the consciousness only that is changed, so the patient is helped to believe in the illusion of being cured without any objective changes happening. The truth is that what we do not control for what we do not know about. It might very well be that we only help the patients subjectively. But then again, what brings the patient to the therapist to begin with is the subjective feeling of being ill, unhappy, or poorly functioning. When the medicine is curing that, the patient is satisfied. It might be that the physician still can have higher ambitions on behalf of the patient, but it can surely be discussed if this is ethical and relevant[86,87].

### CONCLUSIONS

Clinical holistic medicine (psychodynamic short-time therapy complemented with bodywork) seems to be powerful in inducing Antonovsky's salutogenesis (holistic healing) and seems to be highly efficient to heal patients with somatic, mental[88], existential, and/or sexual sufferings; also when former psychiatric/psychological treatment has failed. This clinical study of 109 patients treated in the Research Clinic for Holistic Medicine in Copenhagen during the 2004–2006 period using the square curve paradigm to test the effect of immediate and permanent existential healing documented such an existential healing simultaneously affecting *health, quality of life*, and *general ability to function.* The price for a cure was on average 1,000 EURO, making clinical holistic medicine an efficient, least costly, scientifically based, medical treatment.

The research question we have addressed for more than a decade has been: Would it be possible to create a highly efficient, short-time psychodynamically founded, intervention that could induce salutogenesis and immediate and lasting healing? It seems that clinical holistic medicine as described in a number of papers is able to answer that question positively.

### ACKNOWLEDGMENTS

These studies were supported by grants from IMK Almene Fond. Our research in quality of life has been approved by the Copenhagen Scientific Ethical Committee under number (KF)V.100.2123/91.
